# Microbiota-Derived Metabolite Trimethylamine N-Oxide Protects Mitochondrial Energy Metabolism and Cardiac Functionality in a Rat Model of Right Ventricle Heart Failure

**DOI:** 10.3389/fcell.2020.622741

**Published:** 2021-01-14

**Authors:** Melita Videja, Reinis Vilskersts, Stanislava Korzh, Helena Cirule, Eduards Sevostjanovs, Maija Dambrova, Marina Makrecka-Kuka

**Affiliations:** ^1^Latvian Institute of Organic Synthesis, Riga, Latvia; ^2^Faculty of Pharmacy, Riga Stradiṇš University, Riga, Latvia

**Keywords:** trimethylamine N-oxide, right ventricular dysfunction, monocrotaline, mitochondrial function, cardiovascular diseases

## Abstract

**Aim:** Trimethylamine N-oxide (TMAO) is a gut microbiota-derived metabolite synthesized in host organisms from specific food constituents, such as choline, carnitine and betaine. During the last decade, elevated TMAO levels have been proposed as biomarkers to estimate the risk of cardiometabolic diseases. However, there is still no consensus about the role of TMAO in the pathogenesis of cardiovascular disease since regular consumption of TMAO-rich seafood (i.e., a Mediterranean diet) is considered to be beneficial for the primary prevention of cardiovascular events. Therefore, the aim of this study was to investigate the effects of long-term TMAO administration on mitochondrial energy metabolism in an experimental model of right ventricle heart failure.

**Methods:** TMAO was administered to rats at a dose of 120 mg/kg in their drinking water for 10 weeks. Then, a single subcutaneous injection of monocrotaline (MCT) (60 mg/kg) was administered to induce right ventricular dysfunction, and treatment with TMAO was continued (experimental groups: Control; TMAO; MCT; TMAO+MCT). After 4 weeks, right ventricle functionality was assessed by echocardiography, mitochondrial function and heart failure-related gene and protein expression was determined.

**Results:** Compared to the control treatment, the administration of TMAO (120 mg/kg) for 14 weeks increased the TMAO concentration in cardiac tissues up to 14 times. MCT treatment led to impaired mitochondrial function and decreased right ventricular functional parameters. Although TMAO treatment itself decreased mitochondrial fatty acid oxidation-dependent respiration, no effect on cardiac functionality was observed. Long-term TMAO administration prevented MCT-impaired mitochondrial energy metabolism by preserving fatty acid oxidation and subsequently decreasing pyruvate metabolism. In the experimental model of right ventricle heart failure, the impact of TMAO on energy metabolism resulted in a tendency to restore right ventricular function, as indicated by echocardiographic parameters and normalized organ-to-body weight indexes. Similarly, the expression of a marker of heart failure severity, brain natriuretic peptide, was substantially increased in the MCT group but tended to be restored to control levels in the TMAO+MCT group.

**Conclusion:** Elevated TMAO levels preserve mitochondrial energy metabolism and cardiac functionality in an experimental model of right ventricular heart failure, suggesting that under specific conditions TMAO promotes metabolic preconditioning-like effects.

## Introduction

Impaired energy metabolism is one of the cornerstones of heart failure pathophysiology (Rosca and Hoppel, 2013). Normally, 60–90% of energy is generated through fatty acid oxidation (FAO) (Lopaschuk et al., 2010; Liepinsh et al., 2014); however, in the early stages of heart failure, a shift from fatty acid oxidation toward glucose utilization is observed and is a mechanism of metabolic adaptation (Ventura-Clapier et al., 2011). Compared to healthy patients, heart failure patients exhibit decreased FAO (Dávila-Román et al., 2002), which correlates with cardiac hypertrophy and reduced ejection fraction (Neglia et al., 2007; Byrne et al., 2016). During the progression of heart failure, overall cardiac oxidative metabolism decreases, resulting in energy starvation (Sabbah, 2020). One of the risk factors that lead to disturbances in energy metabolism and further progression of cardiovascular diseases is unhealthy dietary patterns. For instance, high intake of fat, red meat and processed food, as in Western diet, is shown to damage myocardial oxidative capacity, leading to impaired mitochondrial energy metabolism (Neves et al., 2014). Moreover, dietary choices determine the composition of intestinal microbiota, which further unambiguously affects the host metabolism (Lindsay et al., 2020). It has been shown that chronic heart failure is characterized by substantial alterations in gut microbiome composition and reduced microbial variety (Kummen et al., 2018; Mayerhofer et al., 2020). Previous studies suggest a link between the human gut microbiome and the homeostasis of energy metabolism, however, a clear causal relationship between them remains elusive.

In 2011, in a targeted metabolomics study, trimethylamine N-oxide (TMAO) was identified as a metabolite, which is both microbiota- and diet-derived, and associated with the incidence of adverse cardiovascular outcomes (Wang et al., 2011). TMAO is produced in organisms by gut microbiota during the metabolism of common food constituents, such as carnitine, choline and betaine; this process first leads to the production of trimethylamine (TMA), which is subsequently oxidized by the host liver enzyme group called flavin-containing monooxygenases (Janeiro et al., 2018). High intake of TMAO and its precursor has been shown to promote atherosclerosis and exacerbate cardiovascular risks (Wang et al., 2011; Koeth et al., 2013; Ding et al., 2018). A positive correlation between the circulating levels of TMAO and the severity of metabolic syndrome has been observed (Barrea et al., 2018), and elevated TMAO plasma levels were observed in patients with diabetes (Lever et al., 2014; Dambrova et al., 2016). Moreover, in heart failure patients, increased TMAO concentrations correlate with heart failure severity, as shown by NYHA class (Tang et al., 2014, 2015; Trøseid et al., 2015) and heart failure-associated mortality (Suzuki et al., 2016). Although extensive studies of TMAO in various patient populations clearly demonstrate that TMAO can serve as a biomarker, it remains uncertain whether TMAO is directly involved in the pathogenesis of cardiometabolic diseases (Nowiński and Ufnal, 2018).

Diet supplementation with TMAO or its precursors has been shown to exacerbate cardiac dilation, leading to reduced ejection fraction and increased cardiac fibrosis, in an experimental model of heart failure (Organ et al., 2016). In addition, a reduction of circulating TMAO levels by 3,3-dimethyl-1-butanol or iodomethylcholine resulted in attenuated cardiac remodeling after aortic banding (Organ et al., 2020; Wang et al., 2020). On the other hand, the Mediterranean diet, which is focused on regular consumption of TMAO-rich fish and seafoods (Cho et al., 2017), is inversely correlated with fatal coronary heart disease (He et al., 2004) and is proposed to be a strategy for preventing and reducing the risk of cardiovascular and metabolic diseases (Tørris et al., 2014; Widmer et al., 2015; Estruch et al., 2018). Moreover, it was recently shown that chronic treatment with low-dose TMAO was associated with preserved cardiac hemodynamic parameters in Spontaneously Hypertensive rats and Spontaneously Hypertensive Heart Failure rats (Huc et al., 2018; Gawrys-Kopczynska et al., 2020). Such controversial results raise the question of whether increased availability of TMAO plays detrimental or protective roles in the progression of cardiovascular diseases.

It has been shown that both acute and chronic TMAO treatment can cause disruptions in energy metabolism in the heart by impairing pyruvate and fatty acid metabolism (Makrecka-Kuka et al., 2017). However, there is no evidence that TMAO-induced metabolic alterations result in impaired cardiac functionality. It could be hypothesized that long-term TMAO administration could exert preconditioning-like effects, thus improving cardiovascular outcomes after stress conditions, such as hypoxia, pressure overload and altered energy substrate availability. Thus, the aim of the present study was to investigate the effects of long-term TMAO administration in an experimental rat model of monocrotaline-induced right ventricle heart failure. To mimic the chronic increase in TMAO in plasma and tissues, as observed in cases of regular consumption of seafood, TMAO pretreatment for 10 weeks prior to monocrotaline injection was chosen. The effects of the administration of TMAO on indicators of heart failure severity (cardiac functional parameters, heart failure and hypertrophy-related gene and protein expression) and cardiac mitochondrial energy metabolism were studied.

## Materials and Methods

### Experimental Animals

Wistar rats (*n* = 40) weighing 280–380 grams (6–8 weeks old) were obtained from the Laboratory Animal Centre, University of Tartu (Tartu, Estonia) and housed under standard conditions (21–23°C, 12-h light/dark cycle, relative humidity 45–65%) for 2 weeks prior to the start of the experiment. The animals were fed a standard R70 diet (Lantmännen, Stockholm, Sweden) with unlimited access to food and drinking water. All the experimental procedures were performed in accordance with the guidelines reported in the EU Directive 2010/63/EU and in accordance with local laws and policies, and all of the procedures were approved by the Latvian Animal Protection Ethical Committee of the Food and Veterinary Service, Riga, Latvia (Food and Veterinary Service Ethical approval Nr. 105). These studies are reported in accordance with the ARRIVE guidelines (Kilkenny et al., 2010; McGrath et al., 2010). Our previous experiments, in which right ventricular functionality was assessed, indicated that due to interindividual variability, 8–10 animals per group are necessary to obtain significant results; therefore, *n* = 10 per group was chosen. The data from previous experiments in which mitochondrial energy metabolism was studied were subjected to statistical power analysis, and the calculations indicated that the mitochondrial respiration assay requires at least *n* = 5 or 6 per group to produce significant results with a power >0.95.

### Experimental Design

**Figure 1:**
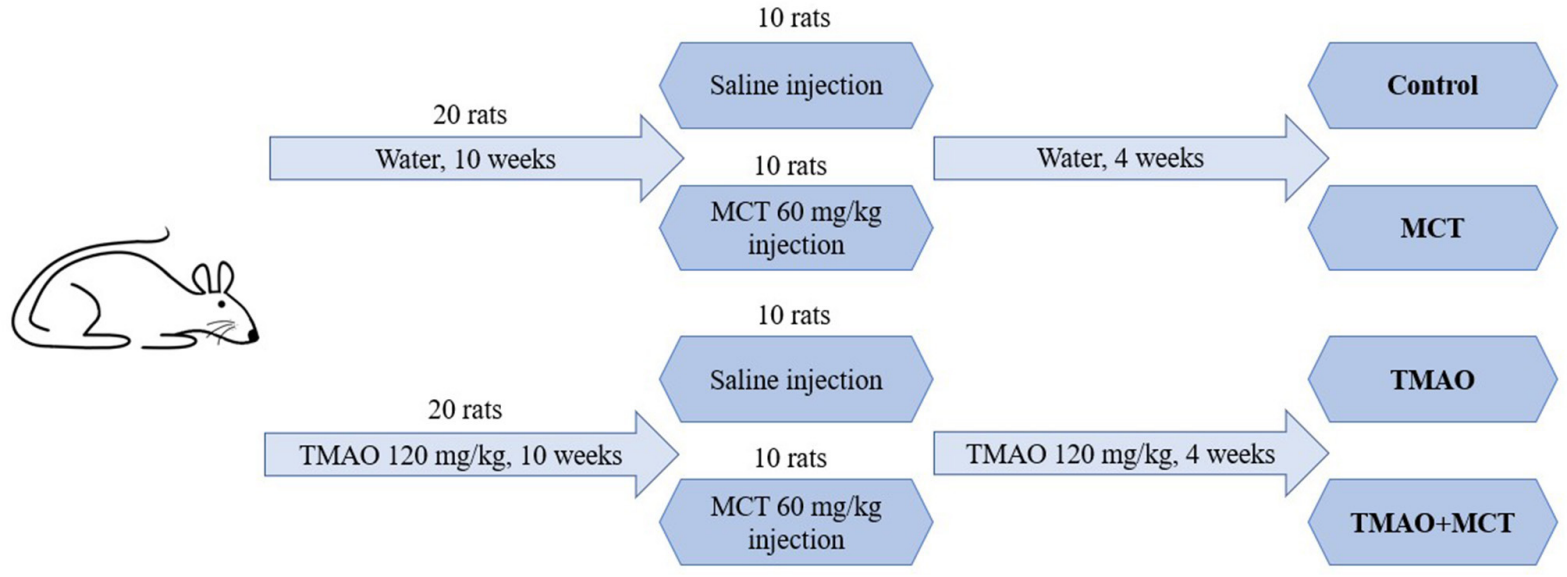
Schematic representation of the study design.

The schematic representation of the study design is shown in Figure 1. The experimental animals were randomly separated into four groups: control (*n* = 10), TMAO (*n* = 10), MCT (monocrotaline) (*n* = 10) and TMAO+MCT (*n* = 10). One animal in the control group and 2 animals in the other groups died during the experiment due to reasons not related to the experimental protocol and treatment. The samples from these rats were excluded from further analysis; therefore, the final animal count was nine rats in the control group and eight rats in the other groups. The animals in the TMAO group and TMAO+MCT group received TMAO (Alfa Aeser, Kandel, Germany) at a dose of 120 mg/kg in their drinking water daily for 10 weeks. To induce pulmonary hypertension and right ventricular remodeling and dysfunction, a single subcutaneous injection of monocrotaline (MCT) (Sigma-Aldrich, Schnelldorf, Germany) at a dose of 60 mg/kg was administered to the animals in the MCT and TMAO+MCT groups. TMAO treatment was continued in both groups that previously received TMAO until the end of the experiment. The rats were weighed twice a week to monitor their general health condition. Since the time from MCT injection to right ventricular failure onset differs markedly (Hardziyenka et al., 2006), a 4-week time point after the administration of MCT was chosen for the echocardiographic assessment of right ventricle functionality based on our pilot experiments in this model. In addition, invasive direct right ventricular pressure measurement was performed. After the assessment of cardiac functionality, the animals were sacrificed, and cardiac tissue and plasma samples were immediately frozen and stored at −80°C for further analysis. In addition, a mitochondrial functionality study was performed using permeabilized cardiac fibers of the right ventricle.

### Echocardiography Assessment and Direct Right Ventricle Blood Pressure Measurement

The rats were anesthetized using 5% isoflurane dissolved in 100% oxygen. After the onset of anesthesia, the concentration of isoflurane was decreased to 2.5%, the experimental animals were placed in a decubitus position, and the chest and upper part of the abdomen were shaved. The animals were connected to a Philips iE33 ultrasonograph (Philips Healthcare, Andover, USA) to record ECG from the II lead. Then, the rat was placed on the left side, and a four-chamber view was recorded from the apical point of view using a Philips (Philips Healthcare, Andover, USA) S12-4 sector array transducer. Right ventricular (RV) end-diastolic area (RV-EDA) and RV end-systolic area (RV-ESA) were recorded. ECG was used to determine the exact time of RV systole and diastole. Furthermore, RV-EDA and RV-ESA were used to calculate RV fractional area change (RVFAC). The rat was again placed in a decubitus position, and functional parameters of the left ventricle were recorded at the papillary muscle level using a Philips (Philips Healthcare, Andover, USA) linear L15-7io transducer.

After the echocardiographic assessment of ventricular anatomy and functioning, invasive direct right ventricular pressure measurement was performed. The anesthetized rat was intubated using a 16-G intravenous catheter and mechanically ventilated with 2% isoflurane dissolved in 100% oxygen at a tidal volume of 1.5 ml/100 g. The abdominal cavity was opened, and the diaphragm was incised to expose the pleural cavity. The ribs on both sides of the chest were cut to access the heart. An 18-G needle was connected to a pressure transducer (AD Instruments, Sidney, Australia) and inserted into the cavity of the right ventricle through the apex of the heart. The right ventricular pressure was measured until a stable pressure reading was obtained.

### Measurement of Organ Mass

To calculate the organ-to-body weight indexes, the heart and lungs were excised and weighed. Then, the right ventricle (excluding the septum) was separated from the heart and weighed.

### Measurements of Plasma Biochemical Parameters

To obtain plasma, the blood samples were centrifuged at 1,000 g and 4° for 10 min and then stored at −80°C until further analysis. The levels of triglycerides and total cholesterol in plasma were measured using commercially available kits from Instrumentation Laboratory (Milan, Italy). The level of free fatty acids (NEFA) was measured using a commercially available kit from Wako Chemicals (Neuss, Germany). All measurements were carried out according to the manufacturer's instructions.

### Quantification of TMAO in Plasma and Tissue Samples

Determination of the TMAO concentrations in the plasma and heart homogenate samples was performed by ultra-performance liquid chromatography-tandem mass spectrometry (UPLC/MS/MS) using the positive ion electrospray mode as previously described (Dambrova et al., 2013; Grinberga et al., 2015). Briefly, obtained tissues were homogenized with water in OMNI Bead Ruptor 24 (Camlab, Cambridge, United Kingdom) at a w/v ratio of 1:10. The obtained homogenates were centrifuged at 20,000 g for 10 min at 4°C. The supernatants were collected and stored at −80°C until further analysis.

Sample preparation was performed by deproteinization with an acetonitrile–methanol mixture (3:1, v/v). The samples were then vortexed and centrifuged at 15,000 g for 20 min. The supernatant was transferred to UPLC vials and used for UPLC/MS/MS analysis. MassLynx 4.1. software with a QuanLynx 4.1. module (Waters, Milford, USA) was used for data acquisition and processing.

### Measurements of Tissue Brain Natriuretic Peptide (BNP)

A Rat BNP 45 ELISA Kit (Abcam, Cambridge, United Kingdom, ab108816) was used to test the levels of makers of congestive heart failure in right ventricular tissue extracts. Extract preparation and analysis were carried out according to the manufacturer's instructions.

### Isolation of RNA and qPCR Analysis

Total RNA was isolated from right ventricular tissues using TRI reagent (Sigma, St. Louis, MO, USA) according to the manufacturer's recommended protocol. First-strand cDNA synthesis was performed using the High-Capacity cDNA Reverse Transcription Kit (Applied BiosystemsTM, Foster City, CA, USA) following the manufacturer's instructions. The qPCR mix consisted of SYBR® Green Master Mix (Applied BiosystemsTM, Foster City, CA, USA), synthesized cDNA, and forward and reverse primers specific for VCP, BNP, αMHC, and βMHC. These genes were chosen to characterize heart failure severity and cardiac hypertrophy. The reaction was carried out in an Applied Biosystems Prism 7500 instrument according to the protocol provided by the manufacturer. The relative expression levels of each of the genes of interest were calculated with the ΔΔCt method and were normalized to the expression level of the VCP gene. The primer sequences used for the qPCR analysis are available in Supplementary Table 1.

### Measurements of Mitochondrial Respiration in Permeabilized Cardiac Fibers

Mitochondrial function was assessed in permeabilized cardiac fibers from the right ventricle that were prepared as previously described (Kuka et al., 2012). The mitochondrial respiration measurements were performed in MiR05 media (110 mM sucrose, 60 mM K-lactobionate, 0.5 mM EGTA, 3 mM MgCl_2_, 20 mM taurine, 10 mM KH_2_PO_4_, 20 mM HEPES, pH 7.1, 0.1% BSA essentially free of fatty acids) at 37°C using an Oxygraph-2k (O2k; Oroboros Instruments, Innsbruck, Austria). Mitochondrial functionality measurements were performed using a previously described respirometry protocol (Makrecka-Kuka et al., 2020). Briefly, palmitoylcarnitine (PC) and malate (10 μM and 0.5 mM, respectively) were used to measure FAO-dependent mitochondrial respiration (F(N)-pathway) in a substrate-dependent LEAK (L) state. Then, ADP was added to a concentration of 5 mM to initiate oxidative phosphorylation-dependent respiration (OXPHOS state). Next, pyruvate (5 mM, complex I substrate, N-pathway) was added to reestablish FN-pathway-linked respiration. Succinate (10 mM, complex II substrate, S-pathway) was added to reconstitute convergent FNS-linked respiration. Then, rotenone (0.5 μM, complex I inhibitor) and antimycin A (2.5 μM, complex III inhibitor) were added to determine the S-linked respiration and residual oxygen consumption (ROX), respectively.

To determine the contribution of each substrate to the respiration rate, the flux control factor was calculated as follows:

$$\begin{array}{l} 1-\frac{Resp.rate\;before\;the\;addition\;of\;substrate}{Resp.rate\;after\;the\;addition\;of\;substrate}. \end{array}$$

To determine the mitochondrial mass in the heart, the citrate synthase activity in tissue homogenates was measured spectrophotometrically as previously described (Srere, 1969).

### Statistical Analysis

The statistical analysis of the data was performed using GraphPad Prism (GraphPad, Inc., La Jolla, USA) software. All the data are represented as the mean ± standard error of the mean (SEM). Data distribution was determined using Shapiro-Wilk's normality test. The statistical significance of the experimental results was verified by one-way ANOVA followed by Dunnett's multiple comparison test (to compare each experimental group to the control group) following an unpaired *t*-test (to compare the MCT group to the TMAO+MCT group). If the data were not normally distributed, the Kruskal-Wallis test followed by Dunn's multiple comparison test was used. The results were considered statistically significant if the *p*-value was < 0.05.

## Results

### Effects of Long-Term TMAO Administration on Heart Failure Severity

**Figure 2:**
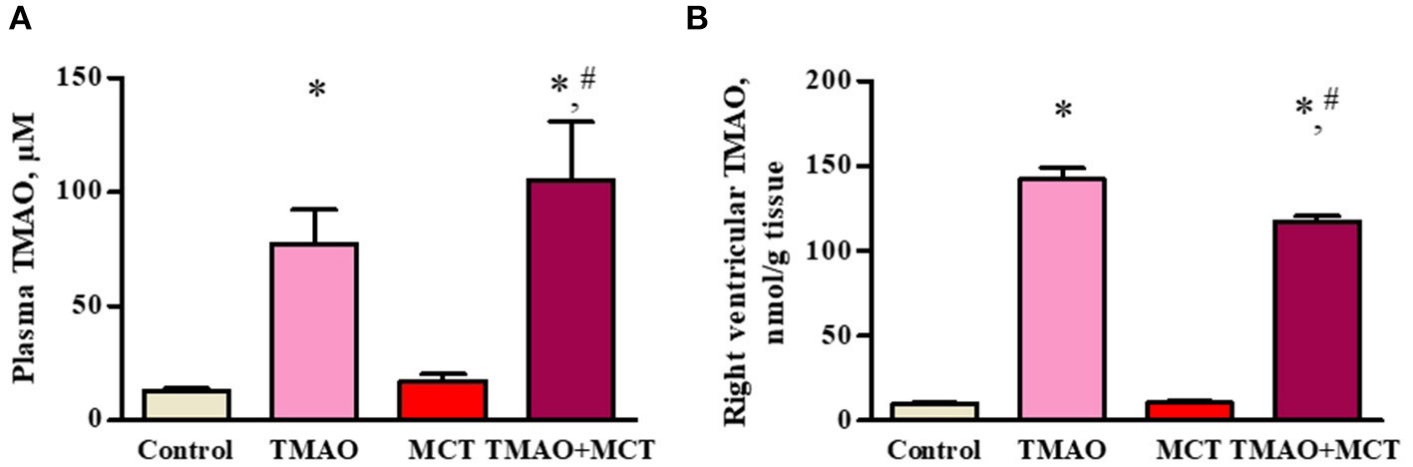
TMAO concentration in plasma **(A)** and right ventricular tissue **(B)** after administration of TMAO at a dose of 120 mg/kg in the drinking water for 14 weeks. The results are presented as the mean ± SEM of 8–9 animals. * Indicates a significant difference from the control group (one-way ANOVA followed by Dunnett's post-test), ^#^ indicates a significant difference from the MCT group (unpaired *t*-test), *p* < 0.05.

Administration of TMAO at a dose of 120 mg/kg in the drinking water for 14 weeks resulted in a 6-fold increase in the TMAO plasma concentrations (up to 100 μM) in both the TMAO and TMAO+MCT groups (Figure 2A). The analysis of the TMAO content in the tissues of the right ventricle revealed that treatment with TMAO resulted in a 14-fold increase in the TMAO tissue content (up to 140 nmol/g tissue) in both groups that received TMAO (Figure 2B).

The echocardiographic assessment did not reveal any significant differences in cardiac function between the control and TMAO groups. Administration of TMAO at a dose of 120 mg/kg in the drinking water for 14 weeks did not affect direct right ventricular (RV) pressure, RV systolic and diastolic area or RV fractional area change (Table 1) as well as ejection fraction and fractional shortening of the left ventricle (Supplementary Table 2). Compared with the control, administration of monocrotaline induced a significant increase (~50%) in direct right ventricular pressure (Table 1). In addition, dilatation of the right ventricle was observed in the hearts of the animals in the MCT group, as indicated by 34 and 83% increases in the RV diastolic and systolic areas, respectively. Subsequently, the right ventricular fractional area change was significantly decreased in the MCT group compared to the control group. Compared to those in the MCT control group, the direct RV pressure measurement was decreased by 22%, the RV diastolic and systolic areas were decreased by up to 27%, and therefore, the RV fractional area change was increased by 25% in the TMAO+MCT group. None of the measured parameters in the TMAO+MCT group were significantly different from those in the control group. Overall, these results indicate that chronically elevated TMAO levels in plasma and cardiac tissue do not affect cardiac functionality, while long-term TMAO administration preserves myocardial mechanical function in monocrotaline-induced heart failure.

**Table 1:** Echocardiographic assessment of right ventricle functionality after administration of TMAO at a dose of 120 mg/kg for 14 weeks in a monocrotaline-induced model of right ventricle heart failure.

	**Control**	**TMAO**	**MCT**	**TMAO+MCT**
Right ventricular pressure, mmHg	21.9 ± 2	22.7 ± 1.3	33.5 ± 5.3^*^	26.1 ± 1.8
Right ventricular diastolic area, cm^2^	0.37 ± 0.02	0.33 ± 0.02	0.5 ± 0.07	0.4 ± 0.04
Right ventricular systolic area, cm^2^	0.2 ± 0.01	0.21 ± 0.02	0.36 ± 0.07^*^	0.26 ± 0.03
Right ventricular fractional area change, %	46.6 ± 2.6	37 ± 2.8	29.7 ± 4.8^*^	37 ± 5

*The results are presented as the mean ± SEM of 8–9 animals*.

* *Indicates a significant difference from the control group (one-way ANOVA followed by Dunnett's post-test), p < 0.05*.

**Figure 3:**
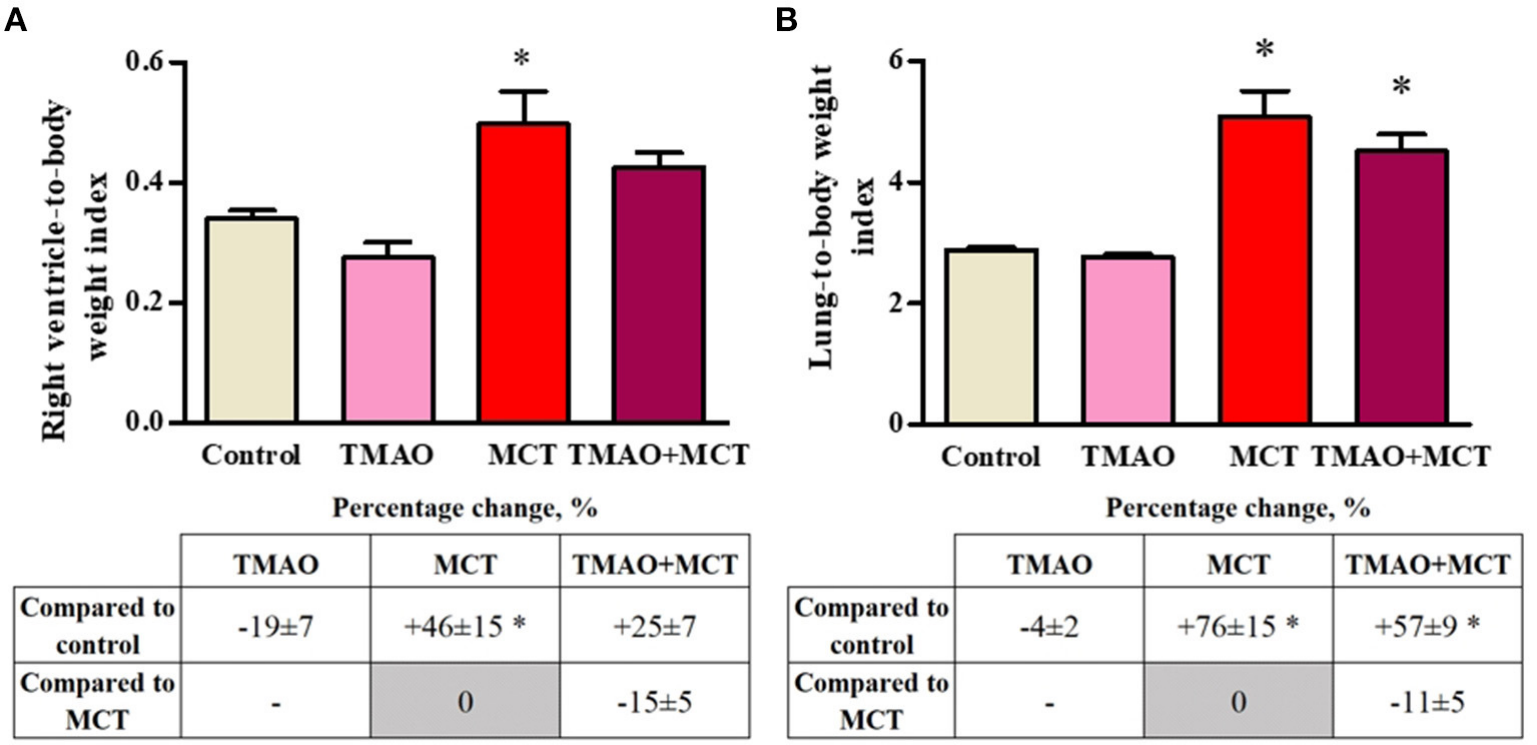
Changes in the right ventricle-to-body weight **(A)** and lung-to-body weight **(B)** indexes after monocrotaline injection. The results are presented as the mean ± SEM of 8–9 animals. * Indicates a significant difference from the control group (one-way ANOVA followed by Dunnett's post-test), *p* < 0.05.

To evaluate MCT-induced cardiac and pulmonary remodeling, the organ mass indexes were calculated. There was no difference in the whole heart-to-body weight index or in the left ventricle-to-body weight index between the experimental groups (Supplementary Figure 1). Long-term TMAO administration did not impact either the right ventricle (Figure 3A) or lung-to-body weight (Figure 3B) indexes. Compared to the control group, the MCT group exhibited increased right ventricle hypertrophy and pulmonary remodeling, as indicated by significant increases in the organ-to-body weight indexes by 46 and 76%, respectively (Figures 3A,B). In the TMAO+MCT group, the right ventricle and lung-to-body weight indexes were decreased by 15 and 11%, respectively, compared to those in the MCT group (Figures 3A,B) suggesting that long-term TMAO administration can partially prevent monocrotaline-induced organ remodeling and hypertrophy.

**Figure 4:**
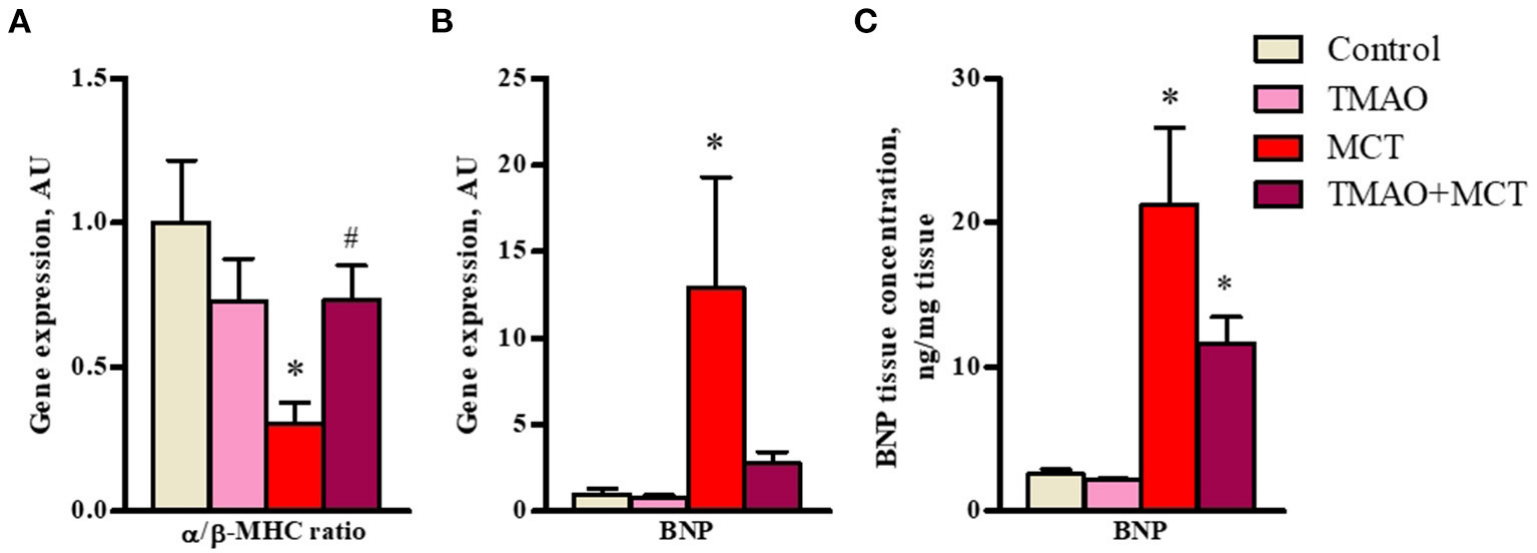
Effect of administration of TMAO at a dose of 120 mg/kg in the drinking water for 14 weeks on heart failure severity-related gene expression **(A,B)** and BNP protein expression **(C)** in right ventricular tissue of a rat model of monocrotaline-induced heart failure. The results are presented as the mean ± SEM of 6–8 animals. * Indicates a significant difference from the control group [one-way ANOVA followed by Dunnett's posttest for **(A)**, Kruskall-Wallis test followed by Dunn's multiple comparison test for **(B)** and **(C)**], ^#^ indicates a significant difference from the MCT group (unpaired *t*-test), *p* < 0.05.

In addition, long-term TMAO administration did not cause any significant changes in the expression of genes related to heart failure and hypertrophy (Figures 4A,B) or in the protein expression of BNP45 (Figure 4C). In the MCT group, a 3-fold decrease in the α*/*β*-MHC* expression ratio (Figure 4A) was observed, indicating a shift in favor of the β isoform caused by right ventricle hypertrophy. In addition, the expression of a maker of heart failure severity, *BNP*, was upregulated by 12-fold in the MCT group (Figure 4B). Consistent with the gene expression results, BNP45 protein expression in cardiac tissue was significantly increased by 10-fold in the MCT group compared to the control group (Figure 4C). In the TMAO+MCT group, the α*/*β*-MHC* expression ratio was 2-fold higher, suggesting less pronounced cardiac hypertrophy compared to that in the MCT group (Figure 4A). The gene and protein expression of BNP was lower in the TMAO+MCT group than in the MCT group (Figures 4B,C). Moreover, measurements of total cholesterol, triglycerides and free fatty acids in plasma did not reveal any significant changes between experimental groups (Supplementary Table 3), therefore our observed effects of TMAO on cardiac function are independent of plasma lipid profile. Overall, these findings indicate that long-term TMAO administration partially reduced the severity of heart failure induced by monocrotaline administration.

### Effects of Long-Term TMAO Administration on Cardiac Mitochondrial Energy Metabolism

**Figure 5:**
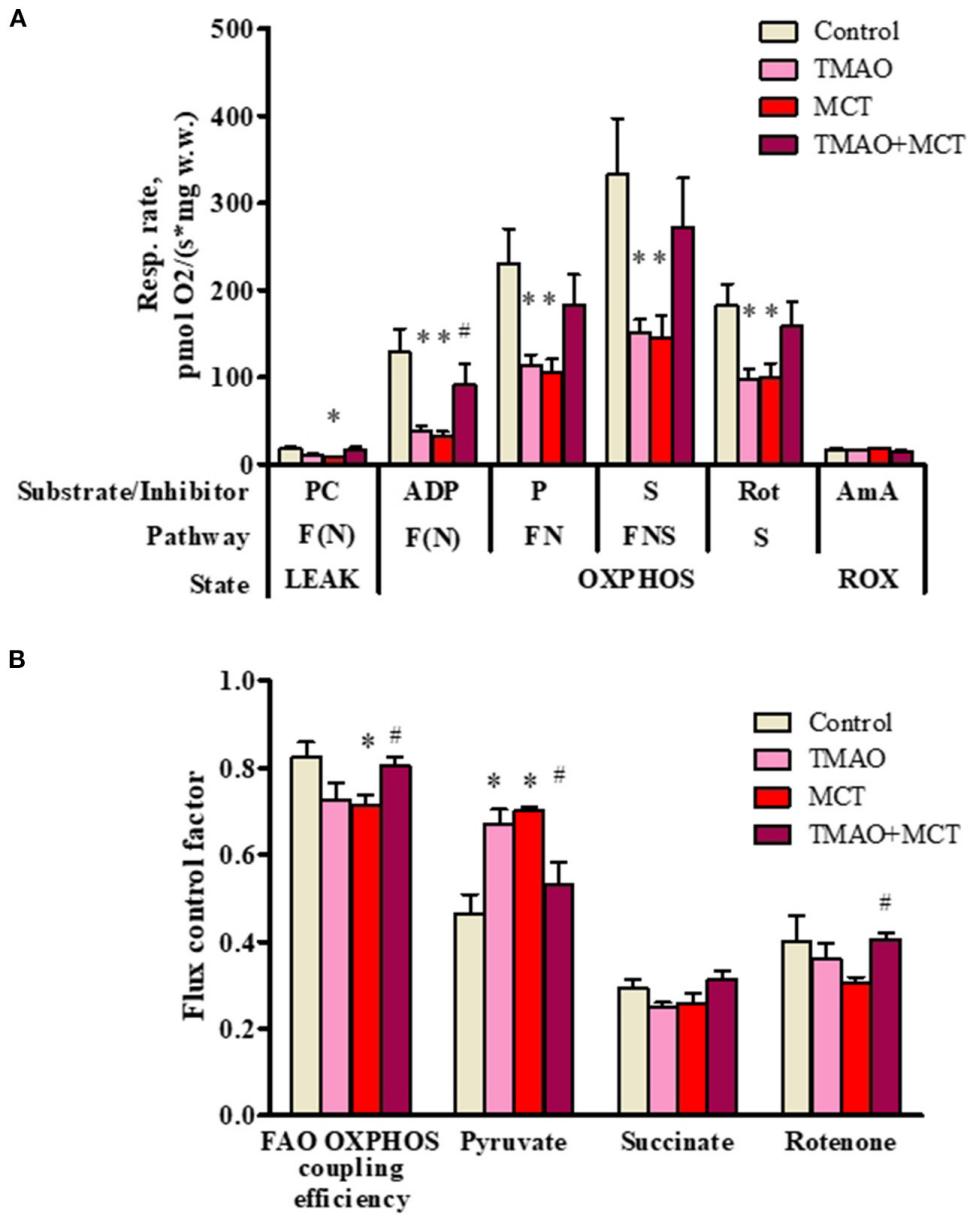
Mitochondrial respiration rate measurements **(A)** and flux control factors **(B)** in right ventricular cardiac fibers using different energy substrates after administration of TMAO at a dose of 120 mg/kg for 14 weeks in a model of monocrotaline-induced right ventricle heart failure. The results are presented as the mean ± SEM of 6 animals. * Indicates a significant difference from the control group (one-way ANOVA followed by Dunnett's post-test), ^#^ indicates a significant difference from the MCT group (unpaired *t*-test), *p* < 0.05. Flux control factor, the contribution of each substrate/pathway to the respiration rate; PC, palmitoylcarnitine; ADP, adenosine diphosphate; P, pyruvate; S, succinate; Rot, rotenone; AmA, antimycin A; F, fatty acid oxidation-dependent pathway; N, NADH pathway; LEAK, substrate-dependent state; OXPHOS, oxidative phosphorylation-dependent state; ROX, residual oxygen consumption.

To further investigate the effects of long-term TMAO administration on energy metabolism, mitochondrial respiration measurements were performed using permeabilized cardiac fibers prepared from right ventricular tissue samples. Long-term TMAO administration decreased the FAO-dependent respiration rate by 69% in the OXPHOS state (Figure 5A), resulting in an 11% decrease in the FAO-dependent OXPHOS coupling efficiency (Figure 5B). Although pyruvate metabolism input to overall respiration was increased by ~44% in the TMAO group, as indicated by Flux control factor analysis (Figure 5B), it was not sufficient to restore FN and FNS pathway-linked mitochondrial respiration in the OXPHOS state (Figure 5A). In the MCT group, there was a 75% decrease in the FAO-dependent respiration rate in the OXPHOS state (Figure 5A) and a subsequent 13% decrease in the FAO-dependent OXPHOS coupling efficiency (Figure 5B). Similar to the TMAO group, in the MCT group, pyruvate metabolism input to respiration was increased by 50% (Figure 5B), but this increase was not sufficient to restore FN- and FNS-pathway-linked respiration in the OXPHOS state (Figure 5A). In contrast to the TMAO group, in the MCT group, the flux control factor for rotenone was reduced (*p* = 0.06), indicating partial complex I dysfunction (Figure 5B). Moreover, in the TMAO+MCT group, mitochondrial energy metabolism was preserved, as shown by normalized respiration rates (Figure 5A), preserved FAO-dependent oxidative phosphorylation efficiency and subsequently decreased pyruvate metabolism input (Figure 5B). Moreover, measurements of the citrate synthase activity (Supplementary Figure 2) in right ventricular tissue showed that there were no differences in mitochondrial mass between the experimental groups. Taken together, the obtained results show that long-term TMAO administration itself induces mitochondrial metabolic preconditioning by causing a switch from fatty acid utilization to pyruvate utilization without affecting electron transfer functionality; moreover, in right ventricle heart failure, TMAO treatment can preserve cardiac mitochondrial energy metabolism.

## Discussion

In the present study, we demonstrate the effects of long-term TMAO administration on cardiac mitochondrial energy metabolism and on right ventricular heart failure progression. Although long-term TMAO administration decreases mitochondrial fatty acid oxidation, it has no adverse effects on cardiac mechanical function. However, unexpectedly, long-term TMAO administration results in preserved mitochondrial energy metabolism, leading to reduced heart failure severity and maintained cardiac functionality. Taken together, these results suggest that elevated TMAO concentrations can exert preconditioning-like effects and exhibit cardioprotective properties.

The role of TMAO as a risk factor in the development of cardiovascular diseases is widely debated around the world. It has been shown that even a 100-fold increase in circulating TMAO levels (up to 60 μM) in rats did not affect cardiac functionality (Ufnal et al., 2014). Similarly, no effects on cardiac parameters in mice were observed after 3 weeks of administration of 0.12% TMAO in the chow (Organ et al., 2016). Although the TMAO concentration in target tissues was not determined in previously mentioned studies, it has recently been shown that TMAO at concentrations up to 10 mM does not affect cell viability, mitochondrial membrane potential or ROS production in rat cardiomyocytes (Querio et al., 2019). Our results complement previous findings, reporting the TMAO level reached in cardiac tissue (up to 140 nmol/g tissue) after 14-week administration of TMAO in drinking water and, thus, provide a rationale behind further dose selection strategies in *in vitro* experiments. In our experimental design, TMAO was administered directly (120 mg/kg in their drinking water) and no further metabolization was needed by gut bacteria. However, the possible interindividual variability caused by alterations in gut microbiota composition should be considered if TMAO precursors (i.e., choline or carnitine) were administered during the experiment, since various bacterial genera are able to metabolize different precursors to synthesize TMA (Jameson et al., 2018). It has also been shown that a retroconversion of TMAO to TMA is possible (Hoyles et al., 2017); however, in our study a low variability of TMAO levels in right ventricular tissue was observed, suggesting that gut microbiota composition and TMA/TMAO production capacity was similar in our experimental animals. Moreover, our results demonstrate that a long-term increase in plasma TMAO levels up to 100 μM and a subsequent increase in TMAO levels in cardiac tissue (up to 140 nmol/g tissue) do not affect cardiac function. In addition, recent studies indicated that TMAO administration does not exacerbate the condition of already present stressors (Querio et al., 2019), such as H_2_O_2_, which is a major contributor to oxidative stress (Nita and Grzybowski, 2016), and doxorubicin, which is known to cause disturbances in cardiac energy substrate metabolism similar to those caused by heart failure (Wu et al., 2016). In our experimental setup, long-term TMAO administration shifted mitochondrial energy substrate utilization from FAO to glucose metabolism, but in contrast to the heart failure group, the TMAO treatment group did not exhibit altered mitochondrial electron transfer system functionality. Since the shift from compensated cardiac hypertrophy to heart failure is preceded by respiratory complex I and II dysfunction (Griffiths et al., 2010), unaltered complex I and complex II function could explain our observations of maintained cardiac functionality even after 14 weeks of TMAO administration, notwithstanding altered energy metabolism. Overall, our findings suggest that despite this metabolic shift, long-term elevations in TMAO levels in plasma and cardiac tissue do not exert detrimental effects on cardiac function.

Previously, increased plasma TMAO levels in experimental models of heart failure led to worsening of cardiac parameters, suggesting that TMAO is a detrimental factor in cardiovascular disease pathophysiology. It has been shown that administration of TMAO and its precursor, choline, exacerbates left ventricle remodeling and cardiac function loss (Organ et al., 2016). Moreover, withdrawal of dietary TMAO even 6 weeks after aortic constriction reversed those changes, indicating an ability of the heart to recover from detrimental changes caused by TMAO (Organ et al., 2020). In addition, a reduction in circulating TMAO levels by 3,3-dimethyl-1-butanol or iodomethylcholine alleviated cardiac hypertrophy and remodeling after aortic banding (Organ et al., 2020; Wang et al., 2020). TMAO-induced impairment in cardiomyocyte contractility and calcium handling (Savi et al., 2018) as well as T-tubule formation (Jin et al., 2020) were suggested as possible mechanisms that may link TMAO to heart failure. In contrast, our results demonstrate that an increase in TMAO levels in plasma and tissues partially prevents the remodeling of the right ventricle and the development of right-sided heart failure. Previously, it has been shown that TMAO treatment reduces cardiac fibrosis and improves cardiac functionality in a model of Spontaneously Hypertensive rats (Huc et al., 2018). Consistent with these findings, our study shows that TMAO administration can partially prevent right ventricular hypertrophy as shown by normalized organ-to-body weight indexes and hypertrophy-related gene expression. Moreover, consistent with our results, protective effects of TMAO were also observed in Spontaneously Hypertensive Heart Failure rats, in which long-term TMAO treatment improved survival and cardiac parameters and lowered plasma NT-proBNP levels (Gawrys-Kopczynska et al., 2020). Similarly, in our study, TMAO administration preserved right ventricular function, as indicated by normalized direct RV pressure and RV fractional area change; moreover, TMAO decreased the expression of a marker of heart failure severity, BNP, in right ventricular tissue. In addition, a recent study showed that administration of betaine, a common TMAO precursor, attenuated pulmonary artery hypertension (Yang et al., 2018). Interestingly, monocrotaline injection was used to induce pulmonary artery hypertension in the previous study; this is the same method we used in our study to induce right ventricle heart failure. Although ~100-fold less TMAO is produced from betaine than from choline (Wang et al., 2014), at least to some extent, the observed protective effects of betaine might be explained by increased bioavailability of TMAO. Overall, previous and present observations suggest that long-term TMAO administration can reduce right ventricular remodeling and improve cardiac function in right-sided heart failure. Moreover, our findings also support the hypothesis that a TMAO-rich Mediterranean diet may prevent and reduce the risk of cardiovascular diseases (Estruch et al., 2018).

The protective role of TMAO was previously explained by its ability to reduce endoplasmic reticulum stress (Makhija et al., 2014), oxidative-nitrative stress and the subsequent vascular and diabetic complications (Lupachyk et al., 2013; Fukami et al., 2015). More recently, some protective effects were attributed to the ability of TMAO to increase diuresis and natriuresis (Gawrys-Kopczynska et al., 2020). In addition, our study proposes preserved cardiac energy metabolism as a possible mechanism underlying the observed protective effects of TMAO. The heart is capable of adapting to both physiological and pathological stressors by shifting from FAO as a dominant energy source to more pronounced utilization of glucose (Brown et al., 2017). In physiological states, this shift could be considered a preconditioning strategy, since the heart is thus better prepared for future stress conditions, such as hypoxia, due to higher reliance on more energy-efficient substrates in the case of oxygen deficiency (Karwi et al., 2018). In the present study previously described metabolic shift was observed, when after long-term TMAO administration FAO was decreased, and pyruvate metabolism was subsequently increased without changes in cardiac functionality. It has been previously shown that TMAO decreases FAO with a mechanism not related to inhibition of carnitine palmitoyl transferase 1 (CPT1) (Makrecka-Kuka et al., 2017). Since we did not observe hindered pyruvate metabolism by accumulation of acylcarnitines, which might occur if carnitine/acylcarnitine translocase (CACT) or CPT2 was inhibited (Chegary et al., 2008; Makrecka et al., 2014; Makrecka-Kuka et al., 2020), most likely the decrease in FAO induced by TMAO administration is not related to direct inhibition of CACT or CPT2. Another possible explanation of our observation that TMAO reduces FAO could be that TMAO indirectly inhibits CACT by a nitric oxide (NO)-dependent mechanism (Tonazzi et al., 2017). However, TMAO is not reported to act as NO donor, moreover, it is shown to downregulate NO production *in vitro* (Sun et al., 2016; Chou et al., 2019) and *in vivo* (Li et al., 2017). Thus, it is unlikely that TMAO could cause indirect inhibition of CACT via NO pathway. Overall, an increase in TMAO concentration appears to induce a metabolic shift, possibly through direct inhibition of β-oxidation, toward more efficient substrate metabolism under stress conditions, such as hypoxia, thus ensuring preserved energy metabolism and subsequently improving cardiac function recovery after injury. Taken together, our results suggest that TMAO administration exhibits cardioprotective properties during heart failure by maintaining metabolic flexibility and preserving fatty acid oxidation, both of which are vital strategies to restore cardiac bioenergetic balance (Kolwicz et al., 2012; Karwi et al., 2018). Moreover, our findings demonstrate that long-term consumption of TMAO-rich foods (i.e., Mediterranean diet) might induce metabolic preconditioning-like effects.

In conclusion, our study presents evidence that chronic TMAO administration protects cardiac functionality by preserving mitochondrial energy metabolism in an experimental model of monocrotaline-induced right ventricle heart failure, where TMAO acts as a preconditioning factor. In addition, our results provide a novel insight on the theory, that the role of TMAO in the pathogenesis of cardiometabolic diseases is not limited to either detrimental or protective effects, suggesting that it might actually be dual and depend on specific conditions.

## Data Availability Statement

The original contributions presented in the study are included in the article/Supplementary Materials, further inquiries can be directed to the corresponding author.

## Ethics Statement

The animal study was reviewed and approved by Latvian Animal Protection Ethical Committee of the Food and Veterinary Service, Riga, Latvia; Food and Veterinary Service Ethical approval Nr. 105.

## Author Contributions

MV, RV, MD, and MM-K performed planning of the study. MV, RV, SK, HC, and MM-K conducted experiments. MV, RV, SK, and MM-K performed data analysis. ES performed bio-analytical assays and data analysis. MV wrote the manuscript with input from RV, MD, and MM-K. All authors have read and approved the final manuscript.

## Conflict of Interest

The authors declare that the research was conducted in the absence of any commercial or financial relationships that could be construed as a potential conflict of interest.
