# Supplementary material for: Microbiota-Derived Metabolite Trimethylamine N-Oxide Protects Mitochondrial Energy Metabolism and Cardiac Functionality in a Rat Model of Right Ventricle Heart Failure
Source: Front Cell Dev Biol. 2021 Jan 14;8:622741. doi: 10.3389/fcell.2020.622741 (PMC7841203; doi:10.3389/fcell.2020.622741)

Supplementary Material

**Table S1.** Gene accession number and primer sequences of qPCR primers.

| **Gene symbol** | **Full name** | **NCBI Accession number** | **Primer sequence** (5'->3') |
| --- | --- | --- | --- |
| **VCP** | Valosin-containing protein | [>NM_053864.2](https://www.ncbi.nlm.nih.gov/entrez/viewer.fcgi?db=nucleotide&id=40254739) | F-AATATTTGACAAGGCACGACAAG |
|  |  |  | R- CCGGTTGGTAGCTCCAATGAT |
| **BNP** | Natriuretic peptide type B | [>NM_008726.5](https://www.ncbi.nlm.nih.gov/entrez/viewer.fcgi?db=nucleotide&id=565671788) | F- TAGCCAGTCTCCAGAGCAATTC |
|  |  |  | R- TTGGTCCTTCAAGAGCTGTCTC |
| **αMHC** | Myosin heavy chain 6 | [>NM_017239.2](https://www.ncbi.nlm.nih.gov/entrez/viewer.fcgi?db=nucleotide&id=186659509) | F- CTCCATCTCTGACAACGCCTATC |
|  |  |  | R- CTCCGGATTCTCCAGTGATGA |
| **βMHC** | Myosin heavy chain 7 | [>NM_017240.2](https://www.ncbi.nlm.nih.gov/entrez/viewer.fcgi?db=nucleotide&id=451958092) | F- GGAGCTGATGCACCTGTAGACA |
|  |  |  | R- AGTGCGGACACGGTCTGAA |

**Table S2.** Ejection fraction and fractional shortening of the left ventricle after administration of TMAO at a dose of 120 mg/kg for 14 weeks.

|  | **Control** | **TMAO** | **MCT** | **TMAO+MCT** |
| --- | --- | --- | --- | --- |
| **Left ventricular ejection fraction, %** | 85±1 | 85±2 | 85±2 | 84±1 |
| **Left ventricular fractional shortening, %** | 49±1 | 50±2 | 50±2 | 48±1 |

The results are presented as the mean ± SEM of 8-9 animals.

**Table S3.** Total cholesterol, triglyceride, and free fatty acid levels in plasma after administration of TMAO at a dose of 120 mg/kg in the drinking water for 14 weeks.

|  | **Control** | **TMAO** | **MCT** | **TMAO+MCT** |
| --- | --- | --- | --- | --- |
| **Plasma total cholesterol, mM** | 1.02±0.05 | 1.06±0.06 | 0.95±0.06 | 1.07±0.07 |
| **Plasma triglycerides, mM** | 1.42±0.18 | 1.41±0.13 | 1.64±0.29 | 1.51±0.09 |
| **Plasma NEFA, mM** | 0.25±0.02 | 0.29±0.03 | 0.31±0.05 | 0.26±0.02 |

The results are presented as the mean ± SEM of 8-9 animals.


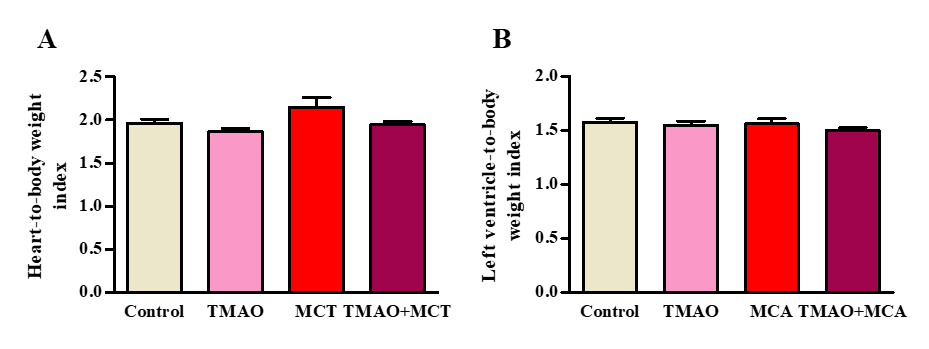
**Figure S1.** Heart-to-body weight **(A)** and left ventricle-to-body weight **(B)** indexes of the experimental animals. The results are presented as the mean ± SEM of 8-9 animals.

**Figure S2.** Cardiac citrate synthase activity in right ventricular tissue homogenates. The results are presented as the mean ± SEM of 6 animals.


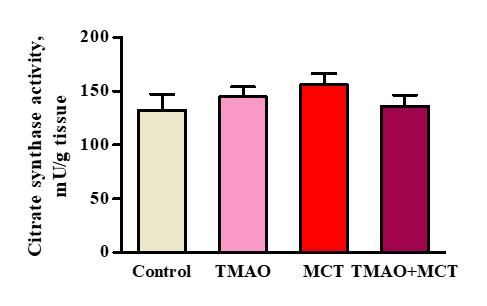

Supplement: Supplementary file 1 [file Data_Sheet_1.docx]
